# Route-planning and the comparative study of future-thinking

**DOI:** 10.3389/fpsyg.2015.00144

**Published:** 2015-02-11

**Authors:** James M. Thom, Nicola S. Clayton

**Affiliations:** ^1^Department of Philosophy, King's College LondonLondon, UK; ^2^Department of Psychology, University of CambridgeCambridge, UK

**Keywords:** mental time travel, navigation behavior, future thinking, chimpanzees, comparative cognition

Research into planning in non-human animals has often focused on anticipation of future needs, with falsification of the “Bischof-Köhler hypothesis” as the gold standard. This hypothesis states that non-human animals are unable to dissociate from the present in order to consider their future drives (Suddendorf and Corballis, 1997). The Bischof-Köhler criterion is intended as a test of constructive future-thinking (or “mental time travel”), in which the future is pre-experienced in the mind's eye (Atance and O'Neill, 2001). Pre-experiencing future scenarios should, the argument goes, allow the motivational drives associated with those scenarios to influence present behavior.

One recent study appears to offer compelling evidence of “route-planning” in chimpanzees (Janmaat et al., 2014). The movements, nesting and feeding behaviors of five wild female chimpanzees were monitored for 275 days. With other factors (e.g., ambient temperature) controlled for, the subjects responded to inter-specific competition for breakfast fruit. The chimpanzees departed toward the breakfast trees earlier when the target fruit was subject to greater competition (typically smaller fruit). Furthermore, the chimpanzees were also more likely to position their nest sites the night before en route to breakfast when breakfast was figs, which were available on the trees for a shorter time than other fruits. The authors argue that this inferred route-planning is a good example of future-thinking outside humans.

However, it is not possible to completely rule out other explanations for the observed behaviors. For example, the authors dismiss time-place associations as an explanation by excluding visits to a breakfast tree after the first. This eliminates a likely learning effect during the course of the experiment, but it cannot account for the accumulated experience of the adult subjects before the start of the 275-day observation. Nonetheless, this work offers intriguing questions for future research. If, as the authors propose, chimpanzees balance predation risk with competition for food when deciding when to depart for breakfast, how flexibly could they respond to new information? If detained for a short time, would they alter course for a tree less likely to be depleted upon arrival?

Route-planning may also be fertile ground for investigation of future-thinking in other non-human animals. Traveling the route to breakfast in one's mind ahead of time certainly seems consistent with a lay definition of future-thinking. Indeed, evidence of neural activity corresponding to navigation of future routes in rats (Pfeiffer and Foster, 2013) has been interpreted by some as evidence of a degree of continuity of future-thinking in mammals (Corballis, 2013), and as a potential constraint on the evolution of future-thinking across other clades (Thom and Clayton, 2014).

Route-planning and constructive future-thinking also exhibit a shared dependence on spatial processing within the hippocampal formation. Neuroimaging studies of constructive future-thinking consistently identify hippocampal activity (see Schacter et al., 2012 for a review), and patients with hippocampal damage have difficulty imagining the future (Klein et al., 2002). However, hippocampal activity is also observed when a subject constructs a scene with no temporal placement at all (Hassabis et al., 2007a). Similarly, some hippocampal patients show impairments in imagination of complex scenes but not simple objects, with specific deficits in “spatial coherence” (Hassabis et al., 2007b, but see Schacter et al., 2012 for a summary of conflicting cases). Maguire and colleagues therefore argue that the hippocampus supports constructive future-thinking by laying down a spatial framework for simulated scenes (Hassabis and Maguire, 2007). Lesion studies likewise identify the hippocampus as important for navigation (Morris et al., 1982). Importantly, activity in the hippocampal formation is also implicated in route-planning: when asked to mentally navigate a route in a scanner, hippocampal activity correlates with the distance between the subject and the end of the route, while activity in adjacent entorhinal cortex corresponds to the Euclidian vector between the subject and her goal (Howard et al., 2014).

We believe that substantive evidence of route-planning that is truly flexible *should* be considered as strongly suggestive of a capacity for a certain type of future-thinking. However, it is not clear that such evidence would be sufficient to refute the Bischof-Köhler hypothesis. Can an intention to arrive at a destination before a certain time be considered a “future need”? More generally, it seems likely that any route-planning to reach breakfast is motivated by current desire for food. The Bischof-Köhler hypothesis has previously been criticized as neither a necessary (Raby and Clayton, 2009) nor a sufficient (Cheke and Clayton, 2010) criterion for constructive future-thinking. Indeed, dissociation of present and future needs is something that even humans are not particularly good at, as evidenced by systematic over-purchasing when shopping while hungry (Nisbett and Kanouse, 1969).

In our view, the Bischof-Köhler hypothesis is too narrow in its focus on anticipation of future needs. It has been used as a litmus test for future-thinking, but future-thinking is not an encapsulated, isolated ability. The Mental Time Travel hypothesis identified several important component processes that overlap with other domains of cognition (Suddendorf and Corballis, 1997), and more recent neuroimaging research has emphasized a shared reliance on hippocampal spatial processing in imagination and future-thinking (Hassabis and Maguire, 2007). It is implausible that any two species should be endowed with identical capacities for prospective cognition, so any pass/fail litmus test will eventually prove to be of limited utility. With deeper elucidation of the mechanisms of human future-thinking, we have the opportunity to search for evolutionary continuity in associated and component processes. To this list we should add route-planning.

## Conflict of interest statement

The authors declare that the research was conducted in the absence of any commercial or financial relationships that could be construed as a potential conflict of interest.

## References

[B1] Atance C. M. O'Neill D. K. (2001). Episodic future thinking. Trends Cogn. Sci. 5, 533–539 10.1016/S1364-6613(00)01804-0 11728911

[B2] Cheke L. G. Clayton N. S. (2010). Mental time travel in animals. Wiley Interdiscip. Rev. Cogn. Sci. 1, 915–930 10.1002/wcs.59 26271786

[B3] Corballis M. C. (2013). Mental time travel: a case for evolutionary continuity. Trends Cogn. Sci. 17, 5–6. 10.1016/j.tics.2012.10.009 23153675

[B4] Hassabis D. Kumaran D. Maguire E. A. (2007a). Using imagination to understand the neural basis of episodic memory. J. Neurosci. 27, 14365–14374. 10.1523/JNEUROSCI.4549-07.2007 18160644 PMC2571957

[B5] Hassabis D. Kumaran D. Vann S. D. Maguire E. A. (2007b). Patients with hippocampal amnesia cannot imagine new experiences. Proc. Natl. Acad. Sci. U.S.A. 104, 1726–1731. 10.1073/pnas.0610561104 17229836 PMC1773058

[B6] Hassabis D. Maguire E. A. (2007). Deconstructing episodic memory with construction. Trends Cogn. Sci. 11, 299–306. 10.1016/j.tics.2007.05.001 17548229

[B7] Howard L. R. Javadi A. H. Yu Y. Mill R. D. Morrison L. C. Knight R.. (2014). The Hippocampus and entorhinal cortex encode the path and euclidean distances to goals during navigation. Curr. Biol. 24, 1331–1340. 10.1016/j.cub.2014.05.001 24909328 PMC4062938

[B8] Janmaat K. R. Polansky L. Ban S. D. Boesch C. (2014). Wild chimpanzees plan their breakfast time, type, and location. Proc. Natl. Acad. Sci. U.S.A. 111, 16343–16348. 10.1073/pnas.1407524111 25349399 PMC4246305

[B9] Klein S. B. Loftus J. Kihlstrom J. F. (2002). Memory and temporal experience: the effects of episodic memory loss on an amnesic patient's ability to remember the past and imagine the future. Soc. Cogn. 20, 353–379 10.1521/soco.20.5.353.21125

[B10] Morris R. Garrud P. Rawlins J. O'Keefe J. (1982). Place navigation impaired in rats with hippocampal lesions. Nature 297, 681–683. 10.1038/297681a0 7088155

[B11] Nisbett R. E. Kanouse D. E. (1969). Obesity, food deprivation, and supermarket shopping behavior. J. Pers. Soc. Psychol. 12, 289–294. 10.1037/h0027799 5821855

[B12] Pfeiffer B. E. Foster D. J. (2013). Hippocampal place-cell sequences depict future paths to remembered goals. Nature 497, 74–79. 10.1038/nature12112 23594744 PMC3990408

[B13] Raby C. R. Clayton N. S. (2009). Prospective cognition in animals. Behav. Processes 80, 314–324. 10.1016/j.beproc.2008.12.005 20522320

[B14] Schacter D. L. Addis D. R. Hassabis D. Martin V. C. Spreng R. N. Szpunar K. K. (2012). The future of memory: remembering, imagining, and the brain. Neuron 76, 677–694. 10.1016/j.neuron.2012.11.001 23177955 PMC3815616

[B15] Suddendorf T. Corballis M. C. (1997). Mental time travel and the evolution of the human mind. Genet. Soc. Gen. Psychol. Monogr. 123, 133–167. 9204544

[B16] Thom J. M. Clayton N. S. (2014). Continuity in hippocampal function as a constraint on the convergent evolution of episodic-like cognition. Cosmology 18, 461–465.

